# Effect of Light Availability on the Interaction between Maritime Pine and the Pine Weevil: Light Drives Insect Feeding Behavior But Also the Defensive Capabilities of the Host

**DOI:** 10.3389/fpls.2017.01452

**Published:** 2017-08-29

**Authors:** Estefanía Suárez-Vidal, Xosé López-Goldar, Luis Sampedro, Rafael Zas

**Affiliations:** Misión Biológica de Galicia, Consejo Superior de Investigaciones Científicas Pontevedra, Spain

**Keywords:** *Hylobius abietis*, *Pinus pinaster*, light availability, herbivory, induced defenses, resin, diterpenes, non-structural carbohydrates

## Abstract

Light is a major environmental factor that may determine the interaction between plants and herbivores in several ways, including top-down effects through changes in herbivore behavior and bottom-up effects mediated by alterations of plant physiology. Here we explored the relative contribution of these two regulation processes to the outcome of the interaction of pine trees with a major forest pest, the pine weevil (*Hylobius abietis*). We studied to what extent light availability influence insect feeding behavior and/or the ability of pines to produce induced defenses in response to herbivory. For this purpose, 3-year old *Pinus pinaster* plants from three contrasting populations were subjected to 6 days of experimental herbivory by the pine weevil under two levels of light availability (complete darkness or natural sunlight) independently applied to the plant and to the insect in a fully factorial design. Light availability strongly affected the pine weevil feeding behavior. The pine weevil fed more and caused larger feeding scars in darkness than under natural sunlight. Besides, under the more intense levels of weevil damage (i.e., those registered with insects in darkness), light availability also affected the pine’s ability to respond to insect feeding by producing induced resin defenses. These results were consistent across the three studied populations despite they differed in weevil susceptibility and inducibility of defenses. Morocco was the most damaged population and the one that induced more defensive compounds. Overall, results indicate that light availability modulates the outcome of the pine–weevil interactions through both bottom-up and top-down regulation mechanisms.

## Introduction

Plants are much more than simple food suppliers for phytophagous insects. When herbivore insects locate and challenge plants an intense two-way interactive flow of information and molecular interactions take place (Mithofer and Boland, 2012; Bruce, 2015). Environmental factors largely influence the outcome of plant–herbivore interactions (Cipollini and Heil, 2010; Karban, 2011). In particular, light availability can be a major modulator of the interaction between plants and insects (Roberts and Paul, 2006; Hua, 2013; Ballare, 2014). Light may directly affect the physiology and the behavior of both the insect and the host, and also indirectly regulate the flow of information between them.

On the one hand, herbivores can use light as a cue for preventing the risk of encounter with predators and parasitoids (Kats and Dill, 1998). Accordingly, many insect herbivores are more active and feed with more intensity on their hosts at night or twilight hours, when dark reduces the risk of being found by their enemies (Fedderwitz et al., 2014). Through influencing the hiding and feeding behavior of insect herbivores, light availability can thus directly modulate the intensity of herbivory, exerting a top-down regulation.

On the other hand, in the last decade, light/dark conditions, day/night cycles, and light quality have been increasingly recognized as regulators of many aspects of physiology of model plants that may affect insect feeding activity through changes in plant attractiveness, palatability, or plant defensive status (Griebel and Zeier, 2008; Ballare, 2009; Ballare et al., 2012; Cerrudo et al., 2012; Goodspeed et al., 2012; de Wit et al., 2013; Kegge et al., 2013). For instance, the emission of volatile organic compounds from plants is largely altered by the night/day cycles and the light/dark physiology (Loughrin et al., 1994; Kegge et al., 2013). Insect herbivores can perceive these changes and use them as risk cues, adapting their behavior accordingly (Maeda et al., 2000; Shiojiri et al., 2006). Thus, light can determine herbivore feeding activity directly through changes in plant physiology exerting a bottom-up regulation.

In particular, light might impact plant responses to herbivory and plant defensive behavior, that is, the ability to increase their effective resistance through the activation of induced resistance mechanisms (Ballare, 2014). Alteration of the hormonal regulation and interferences with herbivore damage signaling cascades, but also constraints in the synthesis of induced defenses due to carbon starvation in absence of photosynthesis may explain the direct role of light as a modulator of plant immune responses (Roberts and Paul, 2006). Plant physiology differs in light and dark and such changes may interfere with the plant hormonal signaling pathways that regulate plant immune responses (Radhika et al., 2010; Kazan and Manners, 2011; de Wit et al., 2013). Particularly, both the jasmonate- and salicylic-dependent resistance of *Arabidopsis* have been reported to be contingent on light availability (de Wit et al., 2013). Accordingly, shade avoiding plants have been found to be more susceptible to pathogens and herbivores than sun-lover species (Moreno et al., 2009; Calder et al., 2011; Cerrudo et al., 2012). Similarly, defensive responses have been also reported to be plastic to light availability (Kurashige and Agrawal, 2005; Calder et al., 2011; Karolewski et al., 2013). For example, *Chenopodium album* plants grown under artificial shade have been shown to be more susceptible to subsequent insect herbivory than counterparts grown under natural light (Kurashige and Agrawal, 2005).

On the other hand, changes in plant primary metabolism and physiology during light/dark cycles directly alter the availability of current photosynthates, potentially affecting the ability of plants for synthesizing induced chemical defenses in absence of light (Guérard et al., 2007; Kangasjarvi et al., 2012; López-Goldar et al., 2016). For instance, the synthesis of terpenes in conifers is known to be constrained in absence of light (Gleizes et al., 1980). Specifically, the production and accumulation of monoterpenes that usually occurs in response to wounding and insect damage can be strongly hampered as monoterpene synthase activity appeared to be severely compromised when light deprivation limits the availability of carbon photosynthates (Lewinsohn et al., 1993; Steele et al., 1995).

Particularly, in the interaction between pine trees and the pine weevil *Hylobius abietis* L. (a major insect pest that causes severe economic losses), previous results have shown that the damage was notably more intense under light deprivation conditions than under natural sunlight (López-Goldar et al., 2016). Other studies also denoted higher activity of the pine weevil during the dark phase of the daily cycle (Fedderwitz et al., 2014). It remains unclear, however, at what extent these results are due to a direct effect of the light on the herbivore feeding behavior, or, instead, they could arise, at least in part, from impaired inducibility of plant defenses in absence of light. The pine weevil is a bark-chewing insect that causes high mortalities by feeding on the phloem and bark of seedlings and young trees of several conifer species (Nordlander et al., 2011). Chemical defenses against this insect are of quantitative nature, that is present in large concentrations with dose-dependent effects on the insect (Moreira et al., 2009; Zas et al., 2014), and are known to be highly inducible after weevil damage recognition (Sampedro et al., 2010; Moreira et al., 2013; Lundborg et al., 2016). Furthermore, induced defenses have been shown to be crucial for weevil resistance (Zas et al., 2011). Based on all these premises we can speculate that potential constrains in the inducibility of defenses in absence of light may be critical for effective resistance against the weevil. Previous results by López-Goldar et al. (2016) showed that concentration of chemical defenses strongly covariated with the intensity of weevil damage, and suggested no detectable effects of prolonged light deprivation on inducibility of chemical defenses. Nevertheless, in that work, light/dark treatments were simultaneously applied to both the plant and the insect, being impossible to disentangle whether the observed responses to the light treatments were mediated by direct effects on the insect behavior or by indirect changes in the plant physiology or both.

**FIGURE 1:**
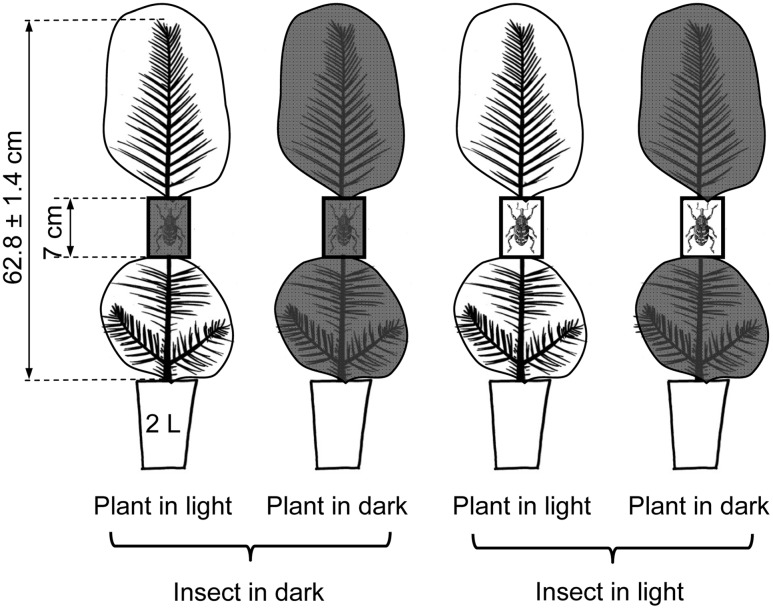
Scheme of the experimental approach showing the factorial application of the light treatments (two levels: complete darkness and natural sunlight) to both the pine and the insects. Plant light treatments were applied by covering the upper and lower parts of the pines with either transparent or completely opaque black plastic bags. Insect light treatments were applied using either transparent or opaque (7 × 4 × 3 cm) cages in which the insects were confined. Two pre-weighted pine weevils (one male and one female) were confined in each cage. Cages were fitted around the stem of the seedlings at approximately half of tree height.

Here we explore the hypothesis that light availability could influence the ability of pine trees for mounting efficient induced defenses in response to herbivory using an experimental approach in which light availability (two levels: darkness and natural sunlight) was independently applied to the plants and to the insects in a full factorial design resulting in four different combinations as showed in **Figure 1**. This approach allowed us to solve the direct and interactive effects of the light availability on both the herbivore and its host. The experiment was done with Maritime pine (*Pinus pinaster* Ait.), the species for which larger effects of the light treatments, and stronger inducibility of defenses were observed in our previous work (López-Goldar et al., 2016). In order to enhance the scope of the study, three Maritime pine provenances originating from contrasting environmental conditions were included in the experimental design.

## Materials and Methods

### Experimental Design

Three-year-old pine trees of three contrasting populations of Maritime pine (*P. pinaster* Ait.) were subjected to 6 days of experimental herbivory with the pine weevil (*H. abietis* L.) under two fully crossed environmental factors, complete light deprivation and natural sunlight applied either to the plant or to the insect (**Figure 1**). The experiment followed a completely factorial design replicated in six blocks, with four different factors: the light deprivation treatment applied to the plant (two levels: complete darkness and natural sunlight), the light deprivation treatment applied to the insect (darkness and sunlight), the herbivory treatment (control and weevil-exposed), and the three pine populations. Due to lack of enough available plants, control plants subjected to the light treatments but not exposed to the herbivore were included in just half of the blocks. Additionally, extra plant material from the three populations (*N* = 6 plants for each population) was sampled just before starting the light and herbivore treatments to have a starting-point reference to evaluate any effect derived from the manipulation of the plants (hereafter manipulation control plants). In total, 126 plants were harvested and sampled for analysis of concentration of plant defenses and non-structural carbohydrates.

### Plant Material and Greenhouse Conditions

Seeds from three Maritime pine populations coming from contrasting environmental conditions (Coastal-Galicia in NW Spain with an Atlantic temperate humid climate with low and short summer droughts, Soria-Burgos in Central Spain with a continental climate with large diurnal and seasonal temperature oscillation and moderate summer droughts, and Riff Mountains in Morocco, with extremely severe summer droughts, Supplementary Figure S1) were sown on 2 l pots filled with peat and perlite (2:1) and fertilized with 16 g of a slow release fertilizer (Granum, Soaga SL, Vilanova de Arousa, Spain; NPK 11-22-9). Plants were grown in a greenhouse with controlled temperature (>14/<24°C night/day) at the Misión Biológica de Galicia (MBG-CSIC, Pontevedra, NW Spain) and watered approximately every week to avoid water stress. In October 2014, when plants were 3 years old and averaged around 70 cm in height, plants were subjected to the light treatments and the experimental herbivory.

### Insects and Herbivory Treatment

Adult pine weevils were captured in a recently clear-felled pine forest (Fornelos de Montes, Pontevedra, Spain; 42°19′34”N, 8°26′18”W) using Nordlander traps baited with ethanol and turpentine (Moreira et al., 2008). Captured weevils were maintained in culture chambers at 10°C and were fed weekly with fresh pine twigs until the start of the experiment.

Two weeks before the experiment began, the central part of the stem of the pines (thereafter named “experimental part”) was prepared for further fitting a wood cage with the insects for the herbivory treatments. In order to avoid injuring the stems and favoring the fitting of the cages, a 7 cm stem section at approximately the mid height of the shoot of the pine trees was delimited, all needles were carefully removed by cutting them at their base, and a 1 cm wide stripe of wiping sponge (Spontex^®^) was carefully fitted around the apical and basal part of the experimental part of the stem using masking tape. At the beginning of the experiment, wood cages (external dimensions 7 × 4 × 3 cm) made with two separable and articulated pieces with transparent acrylic sheets on their larger sides were attached to the stems where the sponges were fitted (Supplementary Figure S2). Two pre-weighted adult pine weevils (one male and one female, previously starved for 24 h at room temperature) were confined into each cage with a small vial providing drinking water (a 1.5 ml Eppendorf tube filled with water and covered with a cotton plug). Control plants not subjected to herbivory were manipulated in the same way but no weevils were confined in the cages.

### Light Treatments

Light treatments on the plants were applied by carefully covering the upper and lower parts of the plant (above and below the experimental cages; **Figure 1**) with either black or transparent 150 μm thick polyethylene bags (Celea SA, Vizcaia, Spain). For applying the light treatments to the insect, the cages in the light deprivation treatment were covered with a black tape and those in the control (natural sunlight) remained with the original transparent acrylic sheets (Supplementary Figure S2). Light treatments (darkness and natural sunlight) were independently applied to the plant and to the insect in a full factorial design resulting in four different combinations as showed in **Figure 1**.

Differences in temperature between light treatments were almost negligible, where mean temperatures at midday were 26.6 ± 1.31 and 25.1 ± 1.23°C inside the dark and transparent bags on the plants, respectively; and 22.7 ± 1.00 and 21.9 ± 0.96°C inside the transparent and dark insect cages, respectively (mean ± SE; *N* = 3; HOBO Pendant^®^data loggers). The transparent polyethylene bags on the plants and the acrylic sheets on the cages slightly reduced the photosynthetic photon flux density to 76.17 ± 1.1 and 71.83 ± 1.3% of that in the greenhouse, respectively (mean ± SE; *N* = 10; Quantum meter MQ-500, Apogee Instruments Inc., Logan, UT, United States). Black bags on plants and black tape on cages completely blocked the incident light (0.0 ± 0.00 μmol m^-2^ s^-1^; mean ± SE; *N* = 10).

### Sampling and Measurements

After 0 (control plants, manipulated but without herbivory) or 6 days of pine weevil feeding on the trees, insects were removed and plants immediately harvested by cutting the shoot just above the pot soil. Then the needles were gently separated from the stem, and the shoot was split into the upper, experimental (the middle part where the pine weevils were allowed to feed) and lower parts. The 7 cm long stem sections immediately above and below the experimental part were stored separately. All fractions were weighed and immediately frozen at -24°C for further chemical analysis.

In order to measure the debarked area in the experimental part of the stem exposed to the pine weevils, the oxidized resin secreted by the bark due to insect wounding activity (mainly oxidized resin acids around the wounds) was previously removed by introducing the stem piece into labeled and pre-weighed borosilicate tubes with 10 ml of methanol for 2 h. Then the cleaned shoot piece was removed, the solvent allowed to evaporate in the fume hood and the tube with the non-volatile resin residue was weighed in a 0.0001 g precision scale. A piece of transparent film was then adhered around the shoot piece, the area of each feeding scar delimited with a permanent marker, the film removed and scanned, and the number and size (mm^2^) of feeding scars in the stem measured with an image analysis software (Image Pro Plus^®^). Total debarked area (mm^2^) in each plant was derived from these measures. The pine weevil may fed on the phloem of pines by opening an initial aperture on the bark and then progressively wounding the margins, thus creating a single large feeding scars, or opening the bark in several places creating several discrete smaller feeding scars.

Then the experimental section of the stem was separated into phloem and xylem using a surgical knife and cut into small pieces. The remaining resin in the plant tissues was extracted with 10 ml of *n*-hexane in pre-weighed borosilicate test tubes for 20 min at 20°C in an ultrasonic bath and then left overnight at room temperature. The extract was transferred into a new pre-weighed borosilicate test tube and the extraction repeated again. Both extracts were mixed and the solvent allowed evaporating to dryness under the fume hood. The wood material was then dried at 65°C and weighed in a 0.0001 g precision scale. The non-volatile resin content in the experimental stem section was estimated gravimetrically as the sum of the dry residual in the methanol (that from the external part of the stem, see above) and hexane extracts, referred to the dry weight of plant material and expressed as milligrams of non-volatile resin per gram of shoot dry weight. The extraction of resin in the upper and lower stem parts (the 7-cm long stem twigs immediately above and below the experimental part) was performed only with *n*-hexane (Moreira et al., 2009) because they have no wounds and no oxidized resin. The analytical procedure was the same than that used for the experimental part.

Non-structural carbohydrates (soluble sugars and starch) were analyzed in the basal part of the stem (just above the root-collar) according to Hansen and Moller (1975). Briefly, soluble sugars were extracted from the shoot tissues (finely grinded in liquid N, 50 mg dry weight) with 1 ml of 80% ethanol in an ultrasonic bath at 80°C for 30 min and centrifuged for 10 min at 2600 *g* in a microcentrifuge. A second extraction was done following the same protocol, the supernatants were mixed, and the concentration of sugars estimated with the anthrone reagent method by measuring absorbance at 630 nm in a microplate reader (Biorad Laboratories Inc., Philadelphia, PA, United States). The pellet was then cleaned with 1 ml of deionized water, digested with 1 ml of 1.1% HCl at 100°C for 30 min, and centrifuged 10 min at 2600 *g*, for starch analysis. The concentration of starch in the solution was measured as above.

### Statistical Analyses

All analysis were carried out fitting mixed models using the PROC MIXED procedure of the SAS System (Littell et al., 2006), assuming light treatments on the plant and the insect, herbivory, population, and all their interactions as fixed factors and block as a random factor. When it was needed, normality was achieved by log-transformations of the dependent variable and residual heterogeneity models across light treatments were used when significant deviations were found. We accounted for the potential effect of weevil size on the damage inflicted on the plants (number of feeding scars, area of each discrete feeding scar, and total debarked area) by including the sum of the individual weights of the two weevils as a covariate in the analyses. Least square means for the main fixed effects and their interactions were derived from these analyses, and statistically compared by a *post hoc* analysis of mean comparisons between all treatments combinations with the PDIFF option of the LSMEANS statement of the PROC MIXED procedure.

## Results

### Weevil Damage

**FIGURE 2:**
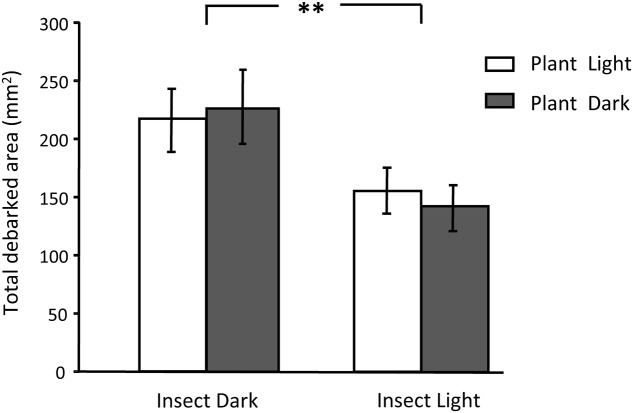
Damage caused by the pine weevil after a 6-day feeding period on 3-year-old Maritime pine trees as a function of the factorial application of light treatments (sunlight/darkness) to the plant and to the insect. Mean ± SE across the three pine populations are shown (*N* = 18). Asterisks denote significant overall differences (*p* < 0.01) between light treatments on the insect.

Light treatment applied to the insect significantly affected the debarked area caused by the pine weevil, which was globally unaffected by the light treatment applied to the plant (**Table 1**). The pine weevil fed more on the pine trees when it was in darkness (light deprivation) (**Figure 2**). The effects of light treatments were consistent across pine populations (no significant interaction, **Table 1**). The size of each feeding scar was also significantly affected by the light treatment applied to the insect but not by that applied to the plant (Supplementary Table S1). In this case, however, the effect of the light treatment on the insect differed among populations (significant interaction, Supplementary Table S1). The mean area of the feeding scars was larger when the insects fed under dark conditions (Supplementary Figure S3a), but these differences were only significant for the Coastal-Galicia population. In relation to the number of feeding scars made by the weevils, a significant interaction between the two treatments was observed (Supplementary Table S1). The light treatment on the insect only affected the number of feeding scars when the plants were in darkness (with higher number of scars when the insects fed at dark), but no significant effects were observed when the plants grew under sunlight (Supplementary Figure S3b).

**Table 1:** Summary of the mixed model for the analysis of the damage caused by the pine weevil on young pine trees of three pine populations after 6 days of feeding under a factorial combination of light availability (sunlight/darkness) applied to the plant and to the insect.

	Total debarked area			
	
Effect	*DF*	*F*	*p* > *F*	
Light on plant (LP)	1,54	0.0	0.859	
Light on insect (LI)	1,54	9.7	**0.003**	
LP × LI	1,54	0.3	0.564	
Pine population (POP)	2,54	3.3	**0.044**	
LP × POP	2,54	0.2	0.814	
LI × POP	2,54	2.1	0.130	
LP × LI × POP	2,54	1.6	0.207	
Weevil weight	1,54	4.0	0.051	


*Degrees of freedom (*DF*), *F* ratios, and associated probability levels are shown. Significant effects (*p* < 0.05) are highlighted in bold font. The size of pine weevils (weevil weight) was included as a covariate in the model.*

**FIGURE 3:**
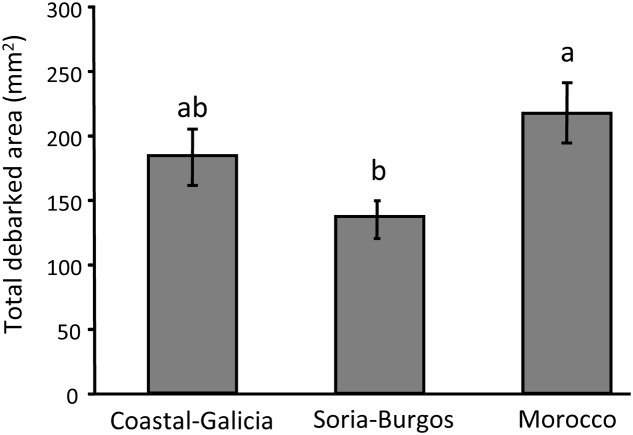
Main effect of pine population on the damage caused by the pine weevil after a 6-day feeding period in 3-year-old Maritime pines. Mean ± SE of total debarked area across the four light treatments are shown (*N* = 24). Different letters above the bars denote significant differences (*p* < 0.05) among populations.

Significant overall differences between the pine populations were also observed for the total debarked area (**Table 1**) and the size of each feeding scar caused by the insect (Supplementary Table S1). Pine weevils fed more intensively on the Moroccan population than on the Soria-Burgos population, with Coastal-Galicia in intermediate position not differing significantly from any of the others (**Figure 3**). Both total debarked area and mean area of the scars covariated positively with the pine weevils’ weight (**Table 1** and Supplementary Table S1).

### Induced Defenses

**FIGURE 4:**
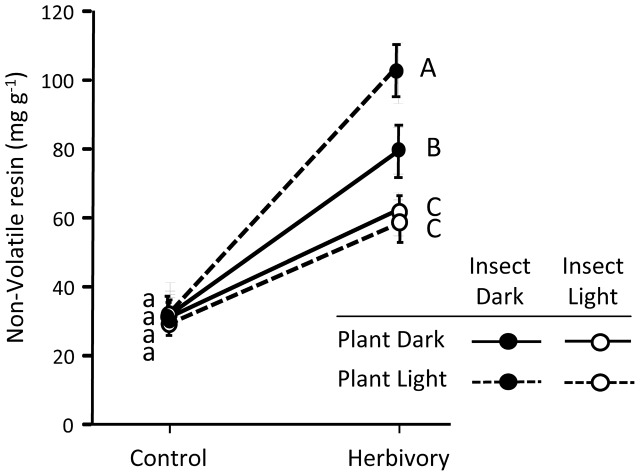
Concentration of pine defenses (non-volatile resin) in control and herbivory-exposed 3-year-old Maritime pine plants under a factorial combination of light availability (sunlight/darkness) applied to the plants and to the insects. Weevils were allowed to feed on the plants for 6 days. Concentration of non-volatile resin was determined in the stem section exposed to the insects (experimental part, see **Figure 6**). Mean ± SE, averaged across the three pine populations are shown (*N* = 9 for control plants and *N* = 18 for weevil-exposed plants). Different lowercase and uppercase letters denote significant differences (*p* < 0.05) between light treatments in control and herbivory-exposed plants, respectively.

Pines strongly responded to weevil feeding damage increasing the non-volatile resin in the stem (significant effect of herbivory in **Table 2**; see also **Figure 4**). This response was significantly affected by the light treatments on the insects (significant insect light treatment by herbivory interaction in **Table 2**). While control plants had similar non-volatile resin levels across light treatments, non-volatile resin after insect herbivory was notably higher when the weevils fed under light deprivation (**Figure 4**). No significant overall effect of the light treatments on the plant was observed on the non-volatile resin after weevil damage (**Table 2**). However, when the insect fed at dark conditions, causing greater damage and stronger plant responses, plants growing under the sunlight accumulated more non-volatile resin after herbivory damage than those growing without light (**Figure 4**), indicating greater inducibility of defenses.

**Table 2:** Summary of the mixed models for the analysis of the concentration of non-volatile resin, soluble sugars, and starch in the stems of young pine trees as affected by the main effects light on plant, light on insect, pine population and herbivory, and their interactions.

		Non-volatile resin		Soluble sugars		Starch		
				
Effect	*DF*	*F*	*p* > *F*	*F*	*p* > *F*	*F*	*p* > *F*	
Light on plant (LP)	1,77	0.6	0.426	11.9	**0.001**	2.5	0.122	
Light on insect (LI)	1,77	9.1	**0.003**	0.0	0.916	0.0	0.914	
LP × LI	1,77	2.2	0.144	1.2	0.274	0.4	0.510	
Herbivory (Herb)	1,77	90.2	**<0.001**	4.6	**0.036**	0.4	0.550	
LP × Herb	1,77	0.5	0.487	3.5	0.066	0.5	0.492	
LI × Herb	1,77	5.9	**0.018**	0.6	0.440	0.2	0.627	
LP × LI × Herb	1,77	0.2	0.626	1.8	0.188	0.6	0.440	
Pine population (Pop)	2,77	3.6	**0.032**	7.5	**0.001**	3.5	**0.035**	
LP × Pop	2,77	1.0	0.387	2.6	0.079	1.8	0.166	
LI × Pop	2,77	0.9	0.418	1.5	0.226	0.6	0.547	
LP × LI × Pop	2,77	1.8	0.180	0.5	0.640	0.3	0.777	
Pop × Herb	2,77	3.3	**0.042**	2.2	0.121	1.0	0.389	
LP × Pop × Herb	2,77	1.6	0.203	0.4	0.656	2.8	0.065	
LI × Pop × Herb	2,77	0.7	0.499	3.6	**0.032**	0.3	0.735	
LP × LI × Pop × Herb	2,77	0.3	0.738	0.9	0.415	0.2	0.792	


*Resin was analyzed in the experimental part of the stem exposed to the weevil; sugars were analyzed in the basal part of the stem. Degrees of freedom (*DF*), *F* ratios, and associated probability levels are shown. Significant effects (*p* < 0.05) are highlighted in bold font.*

**FIGURE 5:**
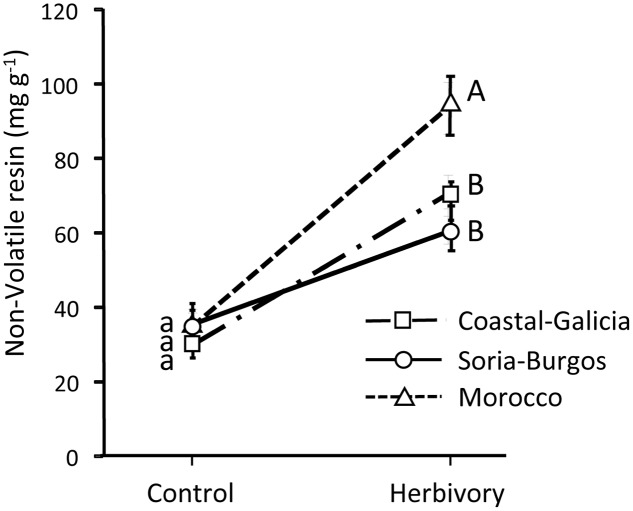
Concentration of pine defenses (non-volatile resin) in control (unexposed) and weevil-exposed 3-year-old Maritime pines of three contrasting populations (Coastal-Galicia, Soria-Burgos, and Morocco). Weevils were allowed to feed on the plants for 6 days. Concentration of non-volatile resin was determined in the stem section exposed to the insects (experimental part, see **Figure 6**). Mean ± SE averaged across the four light treatments are shown (*N* = 12 for control plants and *N* = 24 for herbivory-exposed plants). Different lowercase and uppercase letters denote significant differences (*p* < 0.05) between light treatments in control and herbivory-exposed plants, respectively.

No significant differences in the constitutive concentration of non-volatile resin in the stems were observed among the pine populations (**Figure 5**). However, after weevil feeding, non-volatile resin significantly differed among populations (**Figure 5**), reflecting variation across populations in the inducibility of defenses, also evidenced by the significant population by herbivory interaction (**Table 2**). Inducibility of non-volatile resin was higher in the Moroccan population than in the Coastal-Galicia and Soria-Burgos populations (**Figure 5**).

**FIGURE 6:**
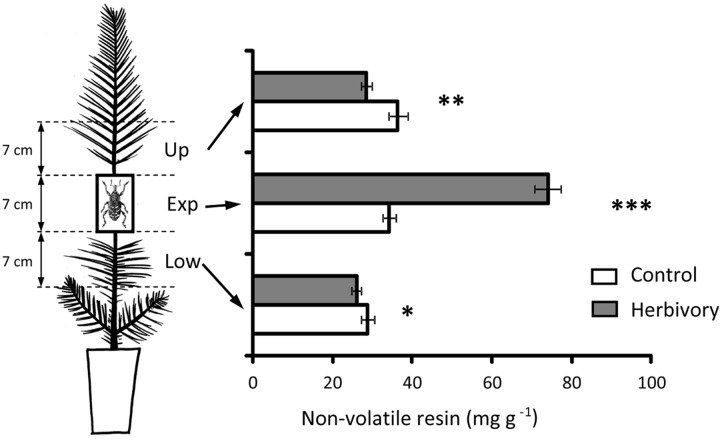
Concentration of non-volatile resin in the upper (Up), experimental (Exp), and lower (Low) parts of the stem of 3-year-old Maritime pine in plants exposed to 6 days of experimental herbivory by the pine weevil and in control plants (non-exposed to the insect). Pine weevils were confined in cages (two weevils per cage) fitted around the experimental part of the stem and allowed to feed on the seedlings for 6 days. Empty cages were fitted to control plants. For each stem part, asterisks above the bars denote significant differences (^∗^*p* < 0.05, ^∗∗^*p* < 0.01, ^∗∗∗^*p* < 0.001) between control and herbivore-exposed plants. Mean ± SE averaged across pine populations and light treatments are presented (*N* = 36 for control plants and *N* = 72 for herbivory-exposed plants).

The increase of non-volatile resin in response to weevil damage was limited to the stem section where the insects fed (**Figure 6**). However, a significant, although comparatively smaller, reduction in the concentration of non-volatile resin in herbivore-exposed plants was observed in the stem sections immediately above and below the experimental section (**Figure 6**).

### Non-structural Carbohydrates

**FIGURE 7:**
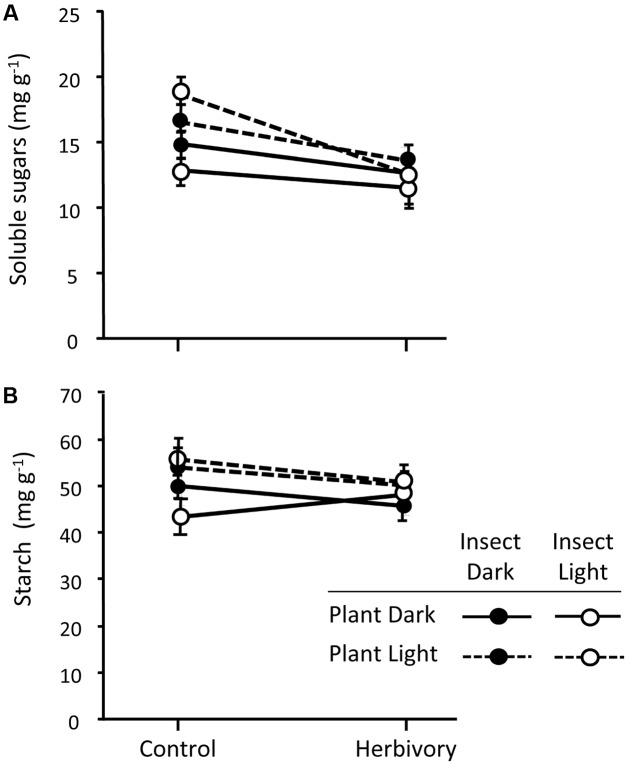
Concentration of soluble sugars **(A)** and starch **(B)** in the basal section of the stem of control and weevil-exposed 3-year-old Maritime pine plants under a factorial combination of light availability (sunlight/darkness) applied to the plant and the insect. Weevils were allowed to feed on the plants for 6 days. Mean ± SE in the basal part of the stems averaged across the three pine populations are shown (*N* = 9 for control plants and *N* = 18 for herbivory-exposed plants). See results of the corresponding mixed models in **Table 2**.

The light treatments applied to the plants significantly affected the concentration of soluble sugars in the basal part of the stem (**Table 2**), with plants growing in darkness having reduced concentration of sugars compared to those growing under natural sunlight (**Figure 7A**). Pine weevil herbivory also affected the concentration of soluble sugars (**Table 2**), with plants exposed to the insects showing lower concentration than control plants (**Figure 7A**). The reduction of soluble sugars after herbivory was similar across all the light treatments (no significant interactions; **Table 2** and **Figure 7A**). Concentration of soluble sugars also varied among pine populations, with higher levels in the Morocco population than in the Soria-Burgos and Coastal-Galicia populations, but the population effect did not interact with the response to the light treatments nor with herbivory (**Table 2** and Supplementary Figure S4a). Reduced concentrations of soluble sugars were found in all the experimental plants (mean = 13.98 ± 1.34, *N* = 108) regarding those levels found in plants sampled before the starting of the light and herbivory treatments (manipulation control plants, mean = 21.38 ± 1.10, *N* = 18), indicating an overall effect of the experimental manipulation on the sugar pool of the plants.

Neither light treatments, nor herbivory, nor plant manipulation significantly affected the concentration of starch in the stem (**Figure 7B** and **Table 2**). Pine populations did however differ in the concentration of starch, with the Moroccan population having greater concentration than the Soria-Burgos and Coastal-Galicia populations (Supplementary Figure S4b).

## Discussion

Results from this study confirm that pine–insect interactions are largely influenced by light availability. The effect of light on the outcome of the interaction was mainly mediated by a direct effect of the light on the insect, whose activity was much higher in absence of light. However, light deprivation to the plants also altered both the weevil feeding behavior and the pine defensive responses to weevil damage, at least in some of the treatment combinations. Results from this paper also revealed that Maritime pine populations significantly differ in their susceptibility to the pine weevil and in their ability to elicit induced responses after weevil damage. However, despite these differences, the responses of the pine seedlings to the different light treatments were highly consistent across populations. Finally, the results clearly indicated that the defensive responses to weevil damage are remarkably localized around the wounding sites, and suggest that the local increase in resin defenses likely involve both the translocation of already existing resin from distal sites, and, putatively, the *de novo* synthesis of chemical defenses upon carbon resources other than current photosynthates.

### Light Availability Influenced Insect Feeding Behavior

The feeding activity of the pine weevils was much more intense when they fed on the pine in absence of light. Both the mean size of the scars and the total debarked area were significantly higher within the opaque experimental cages than within the transparent cages. These results agree with previous findings that reveal maximum mobility and feeding activities of the pine weevils during the night (Merivee et al., 1998; Pszczolkowski and Dobrowolski, 1999; Fedderwitz et al., 2014). Although weevils, as many other insects, are known to follow circadian rhythms (Pszczolkowski and Dobrowolski, 1999), artificial light/cycles are known to be sufficient to trigger consistent feeding behavior patterns in relation to the availability of light (Fedderwitz et al., 2014; López-Goldar et al., 2016). Pine weevils seem, thus, to be more confident under dark conditions, spending more time on each feeding aperture, which results in bigger feeding scars, and thus in greater total debarked area. This result suggests that light availability is affecting pine–insect interactions through top-down regulation processes.

The greater activity of the weevils at dark conditions likely explains the positive effect of the light treatments applied to the insect on the increase of non-volatile resin of the pines in response to the insect damage. Increases of chemical defenses in pine trees in response to wounding or insect damage are usually proportional to the amount of damage suffered: the greater the damage level, the greater the quantitative boost of chemical defenses (López-Goldar et al., 2016). This, indeed, has recurrently complicated the comparison of inducibility across experimental treatments that also affect the amount of insect damage suffered by the plants (Sampedro et al., 2010; Moreira et al., 2013; Lundborg et al., 2016), and it seems to be the case also here as non-volatile resin in weevil-exposed plants was positively correlated with the debarked area caused by the insect (*r*^2^ = 0.23, *p* < 0.001; *N* = 72). This positive correlation between non-volatile resin and weevil damage could lead to think that this trait does not really act defensively against the pine weevil. However, previous studies have consistently shown that this measurement of non-volatile resin correlates well with resistance against the pine weevil, with plants having higher concentration of non-volatile resin being less damaged by the insect in *in vitro* (Moreira et al., 2009), *in vivo* bioassays (Sampedro et al., 2010; Moreira et al., 2013), or at field conditions (Zas et al., 2014). The positive relationship between the amount of damage and the accumulation of non-volatile resin is interpreted, thus, as the consequence of a more intense induced response in the more damaged plants. It should be expected, however, that this increase in non-volatile resin will be translated into a likely reduction of herbivory in the near future.

### Under High Damage Levels, Plants Subjected to Light Deprivation Showed Impaired Inducibility of Defenses

Two different results from this study evidence that light availability for the young pines during herbivory by the pine weevil indirectly conditioned the pine–insect interaction through changes in plant physiology, exerting a bottom-up regulation. On the one hand, despite the light treatments on the plant did not directly affected weevil feeding, they modulated the effects of the insect light treatments on weevil feeding patterns, suggesting that the pine weevil behavior is also regulated by light-driven changes in the plant. Previous studies have shown large changes in weevil feeding behavior depending on the defensive status of the host. For instance, the experimental manipulation of the defensive chemical properties in spruce seedlings have been reported to modify either the feeding rate, the amount of time that the weevils spend feeding on each wound, or the number of wounds they inflicted on the seedlings (Fedderwitz et al., 2016; Lundborg et al., 2016). We can thus speculate that the effect of light availability by the plant on insect feeding behavior is, at least in part, mediated by changes in plant defensive ability.

On the other hand, the inducibility of non-volatile resin in response to weevil feeding was diminished when the plants were deprived from light, although this effect was only seen under high damage levels, that is, when the insects fed in darkness. This finding contradicts previous results which suggested no constraints in the inducibility of pine defenses in response to weevil damage in absence of light (López-Goldar et al., 2016). It should be noted, however, that in the former study light treatments were applied simultaneously to both the plant and the insect, and thus the effect of light deprivation on insect feeding behavior was confounded with that on the plants. Specifically, the potential restriction in the ability to produce induced defenses in absence of light was probably compensated by higher responses due to larger weevil damage at dark conditions (López-Goldar et al., 2016). In the present study we were able to isolate the effect of light availability on the plant from that on the insect. Results show that the increase of non-volatile resin in response to weevil damage was reduced when the plant was deprived from light, but this reduction was only observed when the insect fed at dark conditions causing greater damage. We interpret these results as a consequence of the greater inducibility of defenses when the insects fed more intensively (in darkness). Restrictions to build induced chemical defenses would thus be apparent only when the amount of carbon resources needed for their synthesis is high. We speculate that under high herbivory pressure leading to strong plant induced responses, inducibility of chemical defenses in pine trees could be, at least in part, compromised by light deprivation, and probably by any other environmental factor that affects the photosynthetic activity of the plants (e.g., drought stress through stomata closure). The effects of light to the plant on the inducibility of defenses could be the result of reduced availability of current photosynthates under light deprivation (e.g., Lewinsohn et al., 1993) but also it could be mediated by interferences of light deprivation with the hormonal damage signaling cascades (e.g., de Wit et al., 2013). Further research using isotopic labeled carbon (Guérard et al., 2007) and analyzing changes in hormonal concentrations and gene expression (Kazan and Manners, 2011) would be needed for solving this question.

Additionally, in the study by López-Goldar et al. (2016) weevils were allowed to fed along the whole stem of the plants, and the pine defenses were analyzed in a predetermined stem section irrespective of whether that section was close or not to weevils wounds. Here, on the contrary, we analyzed plant responses to insect feeding just in the same little section of the stem where the weevils were confined and inflicted their damage. Based on the results of the present study we now know that pine defensive responses may drastically differ depending on the proximity to the site of insect damage (see discussion below), so results by López-Goldar et al. (2016) should be managed with caution.

### Genetics of Pine Material Influenced Pine–Insect Interactions

As observed previously in many other plant–insect systems (Yanchuk et al., 2008; Alfaro et al., 2013; Moreira et al., 2013; Mottet et al., 2015), plant genetics largely influenced the interaction between pines and weevils, with pine populations significantly differing in both their susceptibility to the pine weevil and their ability to produce induced defenses in response to feeding damage. Intraspecific variability in susceptibility against the pine weevil has been observed before in other conifer species (Zas et al., 2008; Zas et al., in press), and also in Maritime pine (Zas et al., 2005), although previous studies has focused just on intrapopulation variation aiming to calculate additive genetic variation and the possibilities of breeding for resistance against this important pest. To our knowledge, no attempts have been made yet to explore among-population variation in susceptibility against the pine weevil in any species. However, many conifer species, especially those with isolated and fragmented populations such as Maritime pine, harbor extremely large genetic variation among populations (Grivet et al., 2013). Indeed, population differentiation in Maritime pine is especially large and affects many different adaptive traits such as growth (e.g., Alia et al., 1997), reproductive traits (Santos-Del-Blanco et al., 2012) and also defensive traits (Arrabal et al., 2005; Meijón et al., 2016), and resistance against specific pathogens (Elvira-Recuenco et al., 2014; Zas et al., 2015). Finding significant variation among populations in susceptibility to the pine weevil should be, thus, not surprising. Additionally, the pine weevil has an Eurasian distribution, with its southern limit likely in the northern half of the Iberian Peninsula (Barredo et al., 2015), so not all Maritime pine populations have coevolved with this insect and, thus, some of them could lack specific resistance mechanisms to this pest. Results of the present study agree with this idea, as the population coming from Northern Africa, where no pine weevils naturally exist, was the most damaged by the insect.

Pine populations did not differ in their constitutive levels of non-volatile resin, so other chemical and physical defensive traits should be behind the observed variation in susceptibility against the pine weevil. Previous independent studies have identified several different specific terpenes and phenolic compounds that are relevant for weevil resistance (Lundborg et al., 2016), and that strongly varied among populations (Meijón et al., 2016), but their implication in the observed among-population variation in weevil resistance needs to be confirmed. On the other hand, a significant variation in the non-volatile resin after weevil damage was observed among pine populations, suggesting different abilities to boost induced responses against this insect. However, the variation in the increase of non-volatile resin mirrored the variation in weevil damage, with the Morocco population being the more damaged and the one with the highest response, and the Soria-Burgos population being the less damaged and the less responsive. Thus, as discussed previously, variation among pine populations in inducibility could be a by-product of the variation in susceptibility to the insect.

### Pine Responses to Weevil Damage Were Local but Implied Changes in Distant Tissues

Results from the present study clearly indicated a strong localized response of the young pines to weevil feeding. Non-volatile resin in the experimental part of the stem that was exposed to the insects increased more than twofold after weevil damage. This result is consistent with previous findings demonstrating quick and large responses of young conifers to weevil damage (Sampedro et al., 2010; Lundborg et al., 2016). But the most interesting result regarding the pine responses to weevil feeding was the drastic differences in the induced changes in non-volatile resin depending on the proximity to the site of damage. While non-volatile resin notably increased in the stem section exposed to the weevils, it significantly diminished in the immediately upper and lower stem parts next to the place where the insects fed. These results suggest a rapid translocation of preformed defensive resources to the damage site.

Although the reduction in the concentration of non-volatile resin in the stem sections next to that subjected to weevil damage was comparatively much lower than its increase in the experimental section, the absolute increase of non-volatile resin in the experimental section could all derive from translocation processes whenever the stem sections (and other tissues) farther away also responded similar to those next to the injury site. We lack this information but considering the observed reduction in soluble sugars in the basal stem in response to weevil damage, we can infer that part of those mobilized carbon resources could have been used for *de novo* synthesis of induced defensive compounds. These results are consistent with previous findings in *Populus* sp., in which local-wound responses altered plant-wide patterns of carbohydrate translocation (Arnold et al., 2004; Schultz et al., 2013). These authors found that the carbon sources necessary for the local increase of polyphenolics associated to wounding responses were quite distant from the tissues where the defensive compounds were demanded, requiring translocation of carbohydrates over considerable distances. Sugar depletion upon herbivory can also occur due to hormonal crosstalk, as recently observed in the responses of *Nicotiana attenuata* to simulated herbivory in which reduced leaf sugar concentrations in response to herbivory was mediated by signaling tradeoffs between gibberellin and jasmonate, and not because the storage carbohydrates were used for building induced defensive responses (Machado et al., 2017).

On the other hand, in our study, a decrease in the soluble sugars concentration with light deprivation was observed regardless of herbivory. This result agrees with those reported by Maguire and Kobe (2015), who observed a depletion in non-structural carbohydrate reserves in five temperate tree species under light deprivation. Mobilizing carbon resources in response to carbon limiting stress may be crucial to maintain plant physiological activity and minimize the impact of the stress on plant fitness (Maguire and Kobe, 2015). Overall, both translocation of preexisting defenses and mobilization of stored carbon resources for the synthesis of new defenses are likely occurring in response to weevil feeding.

## Conclusion

Light is an environmental factor regulating plant–herbivore interactions in young pines. Light availability strongly affected the pine weevil feeding behavior, which resulted in consistent greater damage in darkness, thus indicating a marked top-down regulation of pine–insect interactions by light availability. Besides, under great damage by the weevils, inducibility of pine chemical defenses was greater when plants were under natural sunlight conditions. This finding evidence that light availability also affected the response of young pines to herbivory, thus conditioning plant–insect interactions through bottom-up regulation processes too. These results were consistent across the three studied pine populations despite they differed in weevil susceptibility and inducibility of defenses. A long way remains however to understand what physiological mechanisms are behind the observed patterns, and specifically to understand where the resources needed for boosting induced responses to herbivory come from under different environmental situations. Tracing the carbon flow using labeled isotopic carbon sources would help to solve these questions in further research.

## Author Contributions

RZ and LS designed the study and provided the resources required for performing the research; ES-V leaded and all authors performed the experiment; ES-V, XL-G, and LS leaded the chemical analyses and ES-V and RZ the data processing and statistical analyses; ES-V and RZ produced the results and a first draft, and all authors substantially contributed to subsequent revisions.

## Conflict of Interest Statement

The authors declare that the research was conducted in the absence of any commercial or financial relationships that could be construed as a potential conflict of interest.
