# Supplementary material for: Effect of Light Availability on the Interaction between Maritime Pine and the Pine Weevil: Light Drives Insect Feeding Behavior But Also the Defensive Capabilities of the Host
Source: Front Plant Sci. 2017 Aug 29;8:1452. doi: 10.3389/fpls.2017.01452 (PMC5583597; doi:10.3389/fpls.2017.01452)
Supplement: Supplementary file 1 [file Table_1.pdf]

Frontiers in Plant Science 8:1452
doi: 10.3389/fpls.2017.01452 Accepted: 04 August 2017

Effect of light availability on the interaction between Maritime pine and the pine weevil: light drives insect feeding behavior but also the defensive capabilities of the host

Estefanía Suárez-Vidal, Xosé López-Goldar, Luis Sampedro, Rafael Zas*
Misión Biológica de Galicia (MBG-CSIC), Apdo. 28, Pontevedra 36143, Galicia, Spain
* Author for correspondence: rzas@mbg.csic.es

Table S1. Summary of the mixed model for the analysis of the number and size of feeding scars caused by the pine weevil on the stem of young pine trees of three pine populations after 6 days of feeding under a factorial combination of light availability (sunlight/darkness) applied to the plant and to the insect. Degrees of freedom (DF), F ratios and associated probability levels are shown. Significant effects ($p < 0.05$) are highlighted in bold font. The size of pine weevils (weevil weight) was included as a covariate in the model.

		Number of scares		Mean scare area	
Effect	DF	F	Pr > F	F	Pr > F
Light on plant (LP)	1,54	0.3	0.596	0.1	0.789
Light on insect (LI)	1,54	2.4	0.125	4.8	**0.034**
LP x LI	1,54	4.1	**0.049**	1.5	0.219
Pine population (POP)	2,54	2.9	0.066	4.6	**0.014**
LP x POP	2,54	1.8	0.183	1.3	0.293
LI x POP	2,54	0.8	0.437	3.6	**0.033**
LP x LI x POP	2,54	3.1	0.055	1.2	0.305
Weevils weith	1,54	2.3	0.136	14.2	**<0.001**
