# Supplementary material for: Effect of Light Availability on the Interaction between Maritime Pine and the Pine Weevil: Light Drives Insect Feeding Behavior But Also the Defensive Capabilities of the Host
Source: Front Plant Sci. 2017 Aug 29;8:1452. doi: 10.3389/fpls.2017.01452 (PMC5583597; doi:10.3389/fpls.2017.01452)
Supplement: Supplementary file 2 [file Image_1.pdf]

Frontiers in Plant Science 8:1452 **Supplementary material**

doi: 10.3389/fpls.2017.01452 Accepted: 04 August 2017

Effect of light availability on the interaction between Maritime pine and the pine weevil: light drives insect feeding behavior but also the defensive capabilities of the host

Estefanía Suárez-Vidal, Xosé López-Goldar, Luis Sampedro, Rafael Zas*

Misión Biológica de Galicia (MBG-CSIC), Apdo. 28, Pontevedra 36143, Galicia, Spain

* Author for correspondence: rzas@mbg.csic.es

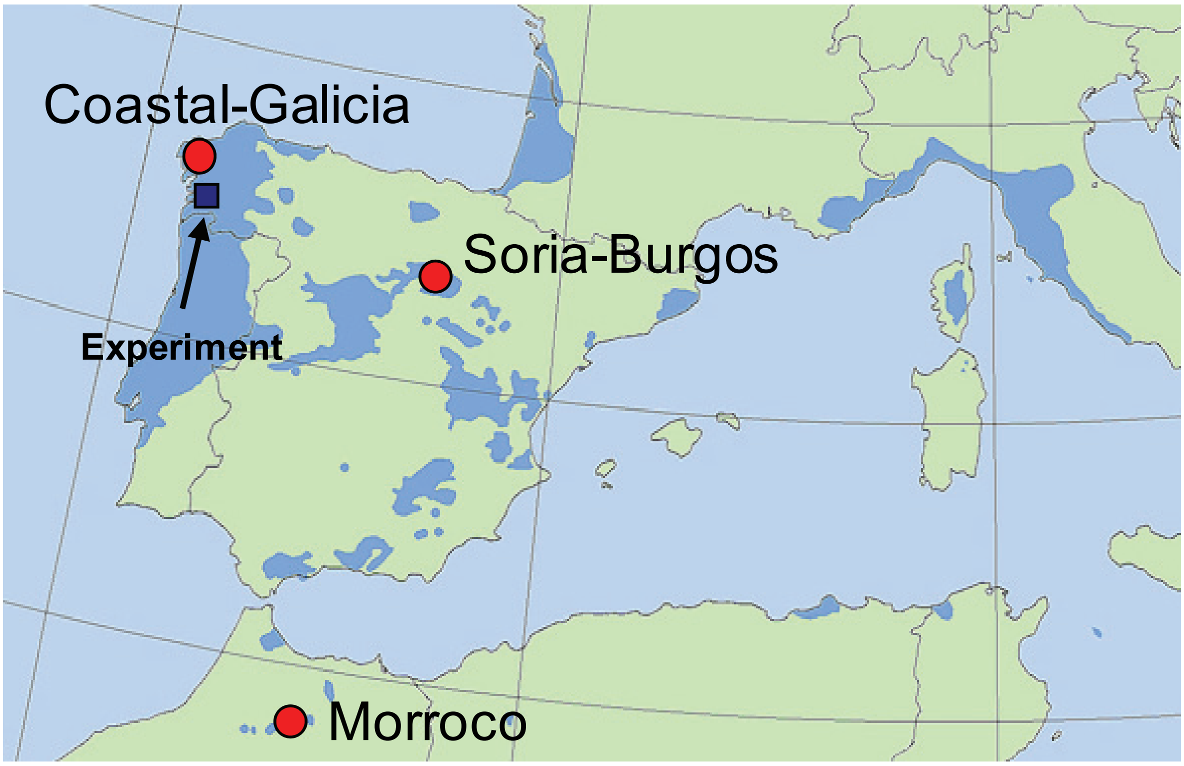

Figure S1. Natural distribution range of the Maritime pine (*Pinus pinaster*, light blue), origin of the pine populations included in the study (red dots) and location of the greenhouse where the experiment was performed (blue square). Distribution map was obtained from EUFORGEN 2009 (www.euforgen.org).
