# Supplementary material for: Effect of Light Availability on the Interaction between Maritime Pine and the Pine Weevil: Light Drives Insect Feeding Behavior But Also the Defensive Capabilities of the Host
Source: Front Plant Sci. 2017 Aug 29;8:1452. doi: 10.3389/fpls.2017.01452 (PMC5583597; doi:10.3389/fpls.2017.01452)
Supplement: Supplementary file 3 [file Image_2.pdf]

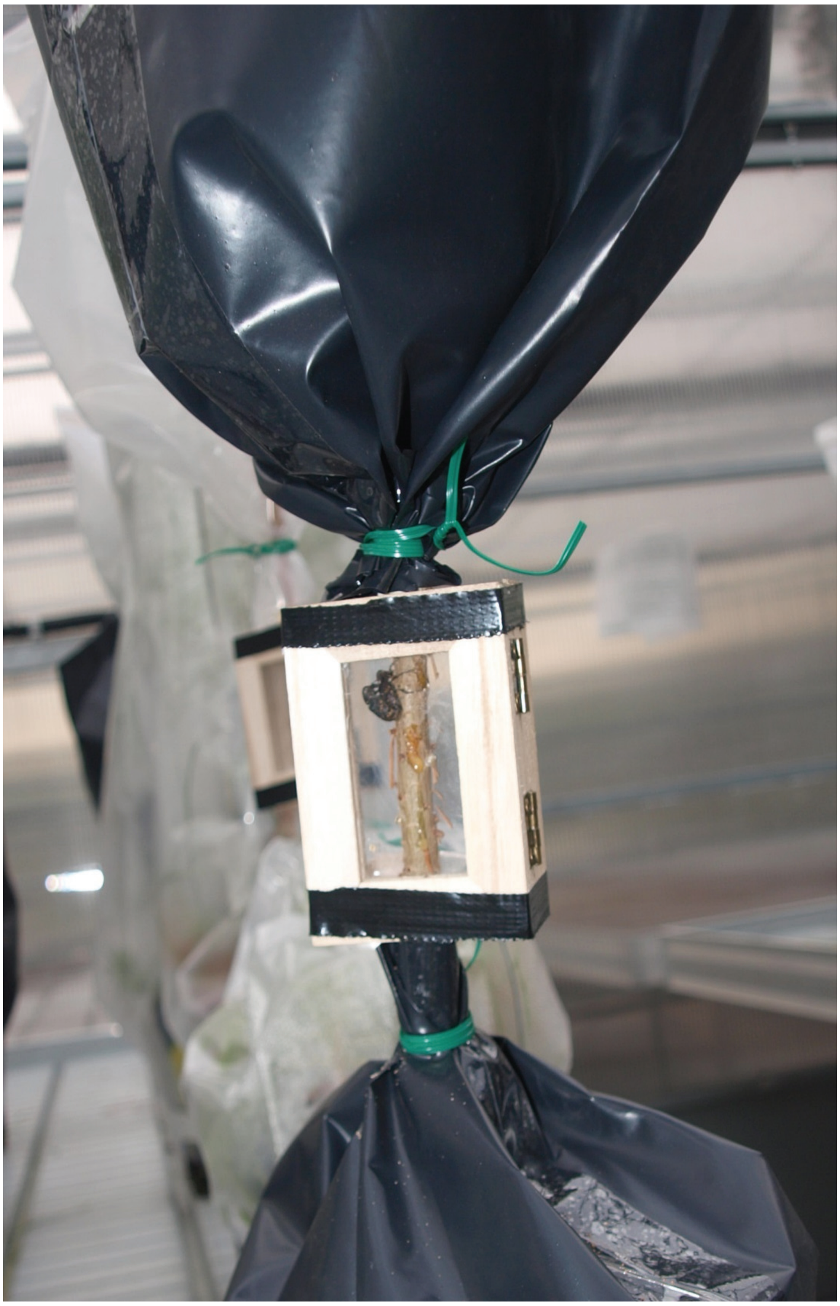

Figure S2. View of a wood cage fitted around the experimental part of the stem with a male and a female pine weevils (*Hylobius abietis*) confined inside the cage. This example corresponds to the combination of natural sunlight treatment on insect (cage with transparent lateral acrylic sheets) and darkness on the plant (black polyethylene bag covering the plant above and below the experimental part of the stem).

Suárez-Vidal et al., 2017. Frontiers in Plant Science 8:1452
doi: 10.3389/fpls.2017.01452 Accepted: 04 August 2017
