# Supplementary material for: Effect of Light Availability on the Interaction between Maritime Pine and the Pine Weevil: Light Drives Insect Feeding Behavior But Also the Defensive Capabilities of the Host
Source: Front Plant Sci. 2017 Aug 29;8:1452. doi: 10.3389/fpls.2017.01452 (PMC5583597; doi:10.3389/fpls.2017.01452)
Supplement: Supplementary file 4 [file Image_3.pdf]

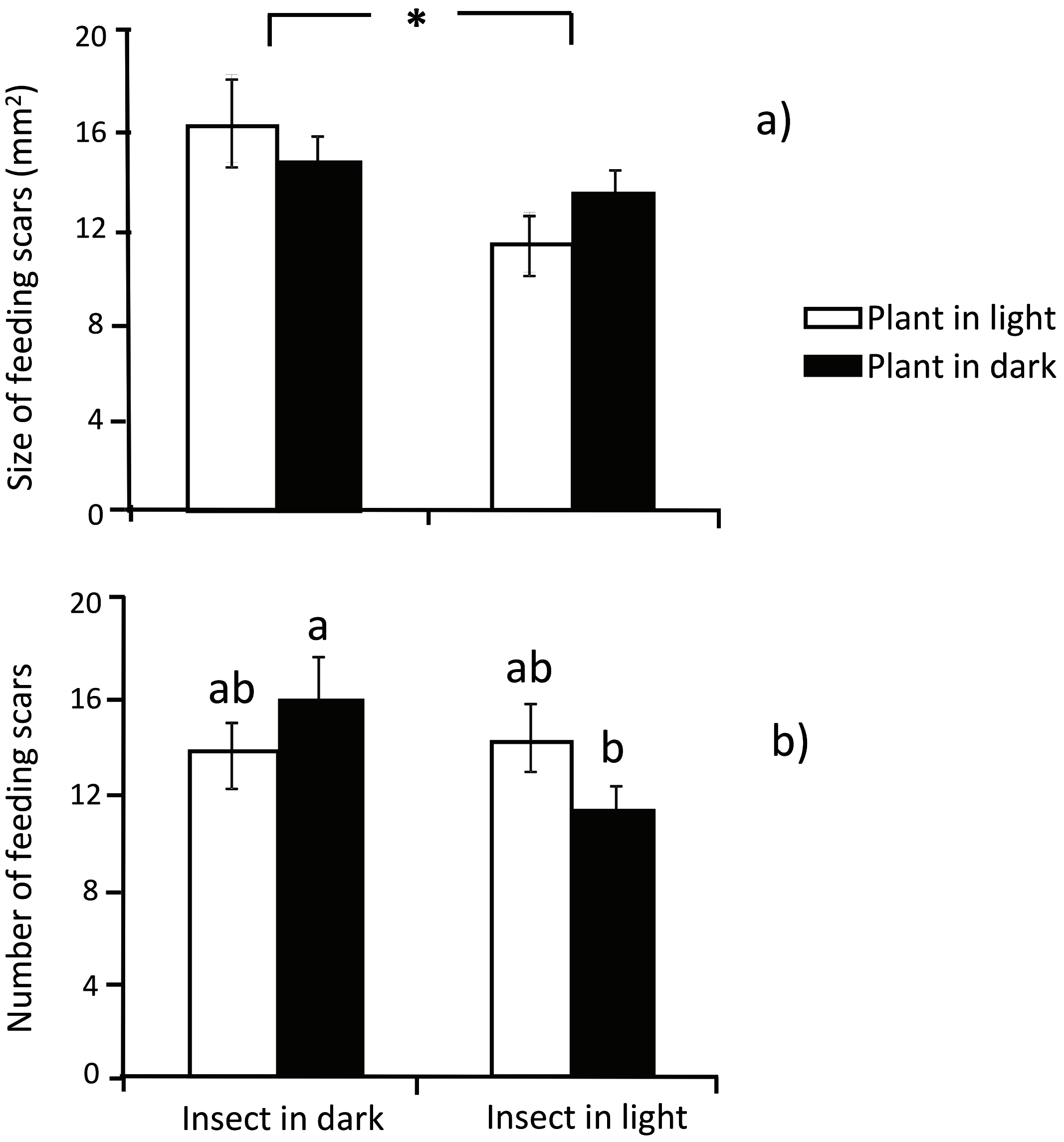

Figure S3. Size (a) and number (b) of the feeding scars caused by the pine weevil on the stem of young pines after 6 days of experimental feeding under a factorial combination of light treatments (sunlight/darkness) applied to the plant and to the insect. The pine weevil may fed on the phloem of pines opening an initial aperture on the bark and then wounding the borders creating a single large feeding scar or opening the bark in several places creating discrete smaller scars. Mean ± S.E across the three pine populations are shown (N = 18). In a) asterisks denote overall significant differences ($p < 0.05$) between insect light treatments, while in b) different letters denote significant differences ($p < 0.05$) among all light treatment combinations.

Suárez-Vidal et al., 2017. Frontiers in Plant Science 8:1452
doi: 10.3389/fpls.2017.01452 Accepted: 04 August 2017
