# Supplementary material for: Effect of Light Availability on the Interaction between Maritime Pine and the Pine Weevil: Light Drives Insect Feeding Behavior But Also the Defensive Capabilities of the Host
Source: Front Plant Sci. 2017 Aug 29;8:1452. doi: 10.3389/fpls.2017.01452 (PMC5583597; doi:10.3389/fpls.2017.01452)
Supplement: Supplementary file 5 [file Image_4.pdf]

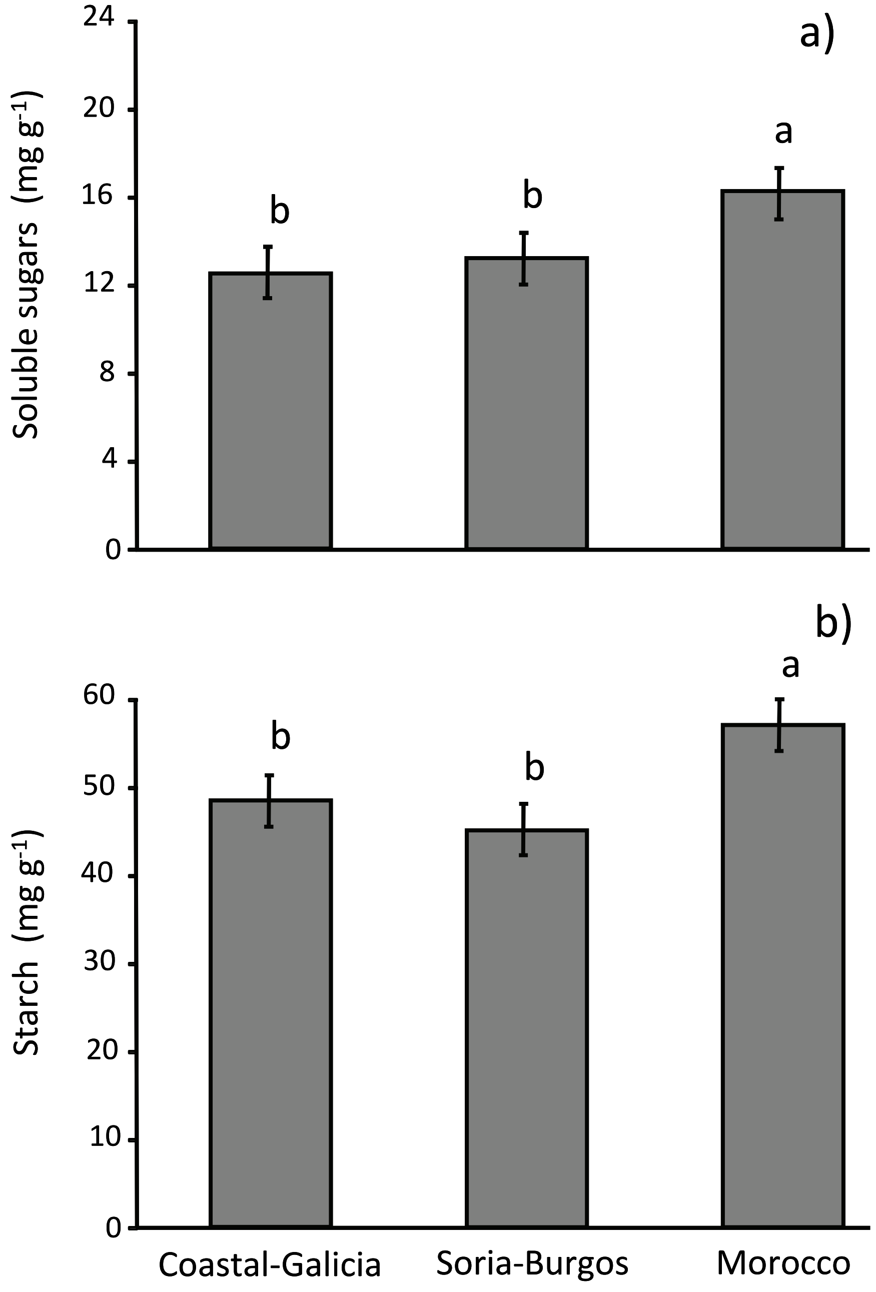

Figure S4. Concentration of a) soluble sugars and b) starch in the basal section of the stem of three Maritime pine populations (Coastal-Galicia, Soria-Burgos and Morocco). Weevils were allowed to feed on the plants for 6 days. Bars are overall means (± S.E) across the four light and the two herbivory treatments (N = 36). Different letters above the bars denote significant differences ($p < 0.05$) among populations.

Suárez-Vidal et al., 2017. Frontiers in Plant Science 8:1452
doi: 10.3389/fpls.2017.01452 Accepted: 04 August 2017
